# Implications of Weight and Body Mass Index for Plasma Donation and Health

**DOI:** 10.5402/2012/937585

**Published:** 2012-11-14

**Authors:** Genna A. Jerrard, Jing Liu, Rosemary C. Case, Mahnaz Motevalli, Stephen G. Bolton, Karen E. King, John Beigel, J. Brooks Jackson

**Affiliations:** ^1^Johns Hopkins Hospital, Carnegie 415, 600 North Wolfe Street, Baltimore, MD 21287, USA; ^2^Clinical Monitoring Research Program, SAIC-Frederick Inc., Frederick, MD 21702, USA; ^3^National Institute of Allergy and Infectious Diseases (NIAID), National Institutes of Health (NIH) Building 10, 8N234-C 10 Center Dr., MSC 1763, Bethesda, MD 20892-1763, USA

## Abstract

This study determined the percentage of potential plasma donors who could donate plasma in the 3 allowable plasma volume limit categories as specified by the Food and Drug Administration (FDA), as well as the association of the body mass index (BMI) of these individuals with age, blood pressure, oral temperature, and pulse. Of 315 plasma donors analyzed, 107 (34.0%) weighed between 110 and 149 lbs (50.0–67.7 kg), 89 (28.2%) weighed between 150 and174 lbs (68.2–79.1 kg), and 119 (37.8%) weighed >175 lbs (79.5 kg), theoretically allowing collection of an additional 101.4 liters (16% more plasma) from both heavier categories based on FDA standards for plasma donor quantities. BMI was positively associated with age, mean arterial pressure (MAP), and pulse (Pearson's *r* = 0.36, 0.24, and 0.18, resp., *P* values <0.05), but not with oral temperature. Average BMI for females was higher than for males (+1.8, *P* = 0.01), and BMI for African Americans was higher than for White and Asian participants (+2.2 and +5.1, resp., *P*s <0.05). A significant association was also found in the sex by race interaction with BMI (*P* = 0.0004). Follow-up analyses suggested a significant difference in BMI by sex among African Americans, higher BMI among African American females than Asian and White males, and higher BMI among White females than African American males (*P*s <0.05).

## 1. Introduction

Site collection of anti-influenza A H1N1 immune plasma as part of an NIAID protocol (IRC001) has revealed lack of knowledge concerning expected plasma volume collections in donor populations. Moreover, although the association between various physiologic parameters has been investigated, no work has detailed the specific relationship between certain physiologic parameters, such as age and vital signs, with body mass index (BMI) in healthy plasmapheresis donors. Our study thus examines these queries in two parts: first, by reporting the percentage of potential plasma donors who could donate plasma in the 3 allowable plasma volume limit categories (see Table 1) as specified by the Food and Drug Administration (FDA) [1]; second, by analyzing the association of the BMI of these individuals with age, blood pressure, oral temperature, and pulse.

## 2. Methods

Individuals aged 18–60 yrs who had received an H1N1 vaccine or reported recovering from an influenza-like illness within 12 months were recruited at the Johns Hopkins Hospital Donor Center from May 2010 to March 2012 for screening as potential plasma donors with no specific mention of weight requirements in recruitment advertising. Eligibility for donation was based on the presence of an anti-influenza H1N1 hemagglutination inhibition (HAI) titer of ≥160 evidenced from a blood sample, along with meeting FDA requirements for plasma donation. Cash compensation was provided to donors for their time for screening and donation visits and was approved by the Johns Hopkins Institutional Review Board and the FDA. Subject charts belonging to the JHU parent study participants who have signed informed consent for the parent study were the source of all data gathered for this substudy.

 Physiologic parameters and personal characteristics (gender, age, race, weight, height, systolic and diastolic blood pressure, pulse, and oral temperature) were collected from records of 315 potential donors (208 females and 107 males) according to the parent clinical study. Values of systolic and diastolic blood pressure, pulse (beats per minute, BPM), and oral temperature (in degrees Celsius) were obtained from one source machine (Dinamap Compact T instrument, Critikon, Tampa, FL, USA), followed by metric measurements of height and weight taken on a stand-on scale (Scale-Tronix 5002, Carol Stream, IL, USA). The latter was performed after participants had removed their shoes, jacket, and all personal items from pockets. To ensure interrater reliability, both devices were routinely calibrated and remained consistent for all screened participants. Personal characteristics of age and race were self-reported by the study participants (racial categories were defined as American Indian, Alaska Native, Asian, Native Hawaiian, Pacific Islander, Black or African American, and White). Later values for BMI and mean arterial pressure (MAP) were calculated using the standard formulas (weight (kg)/[height (m)^2^]) and [1/3 (Systolic BP − Diastolic BP) + Diastolic BP], respectively. The mean and median values of age and vital signs as well as the distribution of sex and race were calculated overall and according to BMI categories (<25 versus ≥25). Pearson's correlation was used to examine the correlation between BMI, age, and vital signs, and chi-square statistics were used to examine the distribution of race and sex by BMI categories. General linear model (GLM) analyses were also performed to examine the independent association between BMI and age, sex, race, MAP, oral temperature, and pulse. Interactions between different demographic categories were also explored. Significant interactions at *P* ≤ 0.10 were included in final GLM analysis. For all analyses, statistical significance was defined as *P* < 0.05. All statistical analyses were performed using SAS Windows 9.2 software (SAS Institute, 2010).

## 3. Results 

Of the 325 individuals screened, physical attributes were available and recorded for 315 individuals. One individual who was screened, but not eligible for the parent study, was 66 years of age. The mean BMI and age of the potential donors were 27 and 35 years, respectively, with 66% being female. More than 32% of potential female donors and 49% of potential male donors weighed 175 lbs or more. Of these 315 subjects, 107 or 34.0% weighed between 110 and 149 lbs (50.0–67.7 kg), 89 or 28.2% weighed between 150 and 174 lbs (68.2–79.1 kg), and 119 or 37.8% weighed 175 lbs (79.5 kg) or more, with each original weight value rounded half up to the nearest integer. Based on parameters set by the FDA, this would theoretically allow the collection of an additional 101.4 liters or 16% more plasma from the two heavier categories based on a donor population whose weight did not exceed the lower weight category. Table 1 shows the uniform set of standards established by the FDA for plasma donor quantities, outlined in the November 4, 1992 memorandum [1–3]. Envisioning a larger plasma donor sample size of 1000 subjects, these percentages would yield local donor values of 340, 282, and 378 in each of the low, middle, and heavy donor categories, respectively.

Analyzing these extrapolated values from known percentages, the expected plasma volumes in a local donor population of 1000 were determined. In the lowest donor weight category, 340 subjects each giving 625 mL of plasma would yield 212500 mL or 212.5 L. In the middle donor weight category, 282 subjects each giving 750 mL of plasma would yield 211500 mL or 211.5 L. Finally, in the heaviest weight category, 378 subjects each giving 800 mL of plasma would yield 302400 mL or 302.4 L. The expected plasma collection volume is therefore 726.4 L in a Baltimore population of *N* = 1000 aged 18–66 years. 

In bivariate correlation analysis, BMI was significantly related to age, blood pressure, and pulse (Pearson's *r* = 0.36, 0.24, and 0.18, resp.), but not oral temperature (Pearson's *r* = 0.01, *P* = 0.81). A comparison of age and vital signs by dichotomized BMI (<25 versus ≥25) suggested that higher BMI was associated with older age, higher systolic and diastolic BP, mean arterial blood pressure, and pulse (*P*s < 0.05). BMI also varied by race, with more African Americans than Whites and Asians in the higher BMI category (*P* < 0.001); however, there was no difference in the distribution of BMI categories by sex (*P* = 0.859).  Table 2 presents the mean age in years, vital signs, race and sex distribution by normal (<25) and above normal (≥25) BMI and overall. Thirteen potential donors had a BMI of 40 or higher, but their blood pressure and pulse were well within donor eligibility requirements for plasma donation.

Preliminary GLM analysis suggested a significant race and sex interaction effect on BMI (*P* < 0.001); therefore, the interaction term was included in the final GLM analysis predicting BMI with age, race, MAP, pulse and oral temperature. Here, age, MAP, and pulse were significant correlates of BMI (*P* values ≤0.05); however, there was no significant association between sex and BMI (*P* = 0.410) or race and BMI (*P* = 0.609) in this model. Further examination of the association between sex, race, and BMI found that African American women (mean BMI = 29.8, SD = 6.2) differ significantly from African American men (mean BMI = 22.5, SD = 4.5). The mean age of the African American men (43 years) and women (42 years) was not statistically different and therefore not a confounding variable, yet African American women had a significantly higher mean BMI of (+7.3) than their male counterparts (*P* < 0.0001). African American female BMI was also significantly higher than that of Asian males (mean = 25.4, SD = 3.2) and White males (mean = 26.4, SD = 4.2), *P* = 0.0415 and 0.0364, respectively. Meanwhile, White females had a significantly higher BMI (mean = 27.5, SD = 6.8) than African American males (*P* = 0.0104).  Table 3 allows a comparison between these adjusted means and the raw means and standard deviations listed by sex and race. Similar patterns are observed relative to the significant differences previously stated between groups. No other differences were observed.

## 4. Discussion

Blood donor population physical characteristics may vary in different regions. It is therefore appropriate to develop predictions for native populations based on local standards. Approximately two-thirds of the adult population interested in plasma donation at our medical center weighed 150 lbs (68.2 kg) or more, potentially allowing greater plasma collection volumes for transfusion or manufacture of high-titer H1N1 intravenous immunoglobulin. Expected plasma volume collection of 726.4 L in a Baltimore population of *N* = 1000 is due to a slightly skewed distribution which favors the heavier weight category. These findings are consistent with other reports showing that 28% of adult, community-dwelling men and women in Baltimore (18 years or older) had a BMI > 29.9 [4]. In such areas, blood donor centers should therefore expect to obtain significantly higher plasma volumes. 

Older age and higher blood pressure were associated with a greater BMI. This has been well documented in other studies [5, 6]. Higher pulse was also associated with greater BMI; however, the fluctuation of pulse as a covariate for BMI between the GLM and linear regression models indicates the uncertainty of its association (*r* = 0.18). Meanwhile, oral temperature was not found to have a significant association with BMI. Our data also revealed an interesting race/sex interaction with BMI where African American women have a significantly higher mean BMI than other studied male racial groups and especially with African American men. Indeed African American men were shown to have a mean BMI less than all other females of the three main racial groups and were the only group whose mean BMI was not overweight, according to NHLBI/NIH standards [7].

Our data reveal that prior determination of the association of race with BMI is dependent on the interaction of sex, as is primarily seen with the difference between female and male African American BMI. Variation in BMI by both sex and racial group has been alluded to in other reports [5, 7]; however, our analysis highlights the additional feature of the importance of the interaction term in this determination. Additionally, our results show female plasmapheresis donors to have a significantly higher BMI than men, which is primarily explained by the difference between male and female African American BMI. 

There were several limitations to our study. Due to a smaller sample size recruited in a local metropolitan area, our conclusions may not be able to be wholly generalized to fit the larger population. Moreover, only a fraction of those screened actually qualified for plasma donation primarily due to a H1N1 titer of <160, which may limit the projected amount of collected plasma from a larger population. For example, there were also four subjects who screen-failed due to high blood pressure. Of these, all four identified as African American and three had weights corresponding to the highest donor weight category. These deferrals due to high blood pressure point out that while recruitment of heavier donors may yield greater plasma volume collections, there will likely also be greater deferrals even though the potential donors with a BMI over 40 in this study had acceptable blood pressure for donation. Therefore, a strategy of recruiting donors based on heavier weight and a lower BMI should be considered in order to maximize plasma volume collected per individual screened. 

Another limitation of our study includes the generalizability to a plasma donor program in the community. Approximately two thirds of our donor population in this study were female which reflects the approximate percentage of the population who work and/or train at our medical center. The majority of donors at most plasma and blood centers in the community are male which would likely result in greater plasma volume collections given males weigh more in general than females.

It should also be noted that racial differences in the prevalence of obesity as defined by BMI should be interpreted cautiously because they do not necessarily correspond to differences in fat mass or percentage of body fat, as BMI does not distinguish fat and lean tissue or represent adiposity directly. Relative to white men and women at the same BMI level, African American men and women tend to have higher lean mass and lower fat mass [8–10]. 

In summary, approximately two thirds of the adult population interested in plasma donation at our medical center weighed 150 lbs (68.2 kg) or more, potentially allowing greater plasma collection volumes for transfusion or manufacture of high-titer H1N1 intravenous immunoglobulin. Older age and higher blood pressure and pulse were associated with greater BMI, while the significant interaction of race and sex with BMI confounded associations between BMI with sex or race alone.

## Figures and Tables

**Table 1:** Volume limits for automated collection of source plasma [1].

Donor weight	Plasma volume or weight	Collection volume
110–149 lbs (50.0–67.7 kg)	625 mL (640 g)	690 mL (705 g)
150–174 lbs (68.2–79.1 kg)	750 mL (770 g)	825 mL (845 g)
175 lbs and up (79.5 kg)	800 mL (820 g)	880 mL (900 g)

**Table 2:** Bivariate analysis of physiological parameters and BMI, stratified by <25 and ≥25 BMI.

	Overall *N* = 315	<25 BMI *N* = 145	≥25 BMI *N* = 170	*P* value
	Mean = 35.1	Mean = 30.2	Mean = 39.3	
Age (years)	Median = 32.0	Median = 26.0	Median = 39.5	**<0.0001**
	Std Dev = 11.9	Std Dev = 9.6	Std Dev = 12.2	

	Mean = 36.5	Mean = 36.5	Mean = 36.6	
Oral temp. (°C)	Median = 36.6	Median = 36.6	Median = 36.6	0.811
	Std Dev = 0.58	Std Dev = 0.6	Std Dev = 0.5	

	Mean = 126.9	Mean = 121.9	Mean = 131.2	
BP systolic (mm Hg)	Median = 127.0	Median = 121.0	Median = 132.0	**<0.0001**
	Std Dev = 16.1	Std Dev = 15.1	Std Dev = 15.7	

	Mean = 73.8	Mean = 72.2	Mean = 75.1	
BP diastolic (mm Hg)	Median = 74.0	Median = 72.0	Median = 76.0	**0.043**
	Std Dev = 9.9	Std Dev = 9.5	Std Dev = 10.2	

	Mean = 91.5	Mean = 88.8	Mean = 93.8	
Mean arterial pressure (mm Hg)	Median = 91.3	Median = 88.3	Median = 93.5	**<0.0001**
	Std Dev = 10.6	Std Dev = 10.1	Std Dev = 10.5	

	Mean = 71.5	Mean = 70.5	Mean = 72.4	
Pulse (beats per minute)	Median = 71.0	Median = 70.0	Median = 73.0	**0.0013**
	Std Dev = 11.4	Std Dev = 11.5	Std Dev = 11.2	

Race				
African American	59	16 (27%)	43 (73%)	
Asian	46	32 (70%)	14 (30%)	**0.0002**
White	200	91 (46%)	109 (54%)	
*Other	10	6 (60%)	4 (40%)	

Sex				
Male	107	50 (47%)	57 (53%)	0.859
Female	208	95 (46%)	113 (54%)	

*The racial category “other” represents all choice races not included among African American, Asian, or White categories.

**Table 3:** Raw means and standard deviations for comparisons of all sex/race groups.

Race	Female			Male		
	*N*	Mean BMI	Std Dev	*N*	Mean BMI	Std Dev
African American	41	31.2	6.19	17	24.6	4.45
Asian	21	24.5	2.25	25	23.9	3.22
White	135	27.2	6.79	61	26.7	4.21
Other	5	23.0	1.04	4	28.9	3.35
